# Supplementary material for: SbcB facilitates natural transformation in Vibrio cholerae in an exonuclease-independent manner
Source: J Bacteriol. 2024 Dec 13;207(1):e00419-24. doi: 10.1128/jb.00419-24 (PMC11784430; doi:10.1128/jb.00419-24)
Supplement: Table S1 — Strains used in this study. [file jb.00419-24-s0002.pdf]

Table S1 - *Strains used in this study*

Strain #*	Genotype	Figures	Use in manuscript
SAD030	*V. cholerae* E7946 Sm^R	Fig. 6B	parent for all strains in this study, and the parent for spontaneous Rif^R mutant frequency assays
TND0905 / SAD2468	P_{tac}-*tfoX*, Δ*luxO*, *lacZ*::*lacI*^{q}, *pilA*^{S67C}, Δ*VC1807*::*Zeo*^{R}	Fig. 1, 3D, 5, 6A, 6C	parent for NT frequency assays and ssDNA exonuclease activity assays
TND4174 / SAD3565	Δ*sbcB*::*Tm*^{R}, P_{tac}-*tfoX*, Δ*luxO*, *lacZ*::*lacI*^{q}, *pilA*^{S67C}, Δ*VC1807*::*Zeo*^{R}	Fig. 1, 3D, 5, 6A	Δ*sbcB* for NT frequency assays
TND4301 / SAD3566	Δ*lacZ*::*Spec*^{R}-P_{native}-*sbcB*, Δ*sbcB*::*Tm*^{R}, P_{tac}-*tfoX*, Δ*luxO*, *pilA*^{S67C}, Δ*VC1807*::*Zeo*^{R}	Fig. 1, 6A	Δ*sbcB lacZ*::*sbcB* for NT frequency assays
TND2245 / SAD3567	Δ*comM*::*Carb*^{R}, P_{tac}-*tfoX*, Δ*luxO*, *lacZ*::*lacI*^{q}, *pilA*^{S67C}, Δ*VC1807*::*Zeo*^{R}	Fig. 3D, 5	Δ*comM* for NT frequency assays
TND4175 / SAD3568	Δ*sbcB*::*Tm*^{R}, Δ*comM*::*Carb*^{R}, P_{tac}-*tfoX*, Δ*luxO*, *lacZ*::*lacI*^{q}, *pilA*^{S67C}, Δ*VC1807*::*Zeo*^{R}	Fig. 3D	Δ*sbcB* Δ*comM* for NT frequency assays
TND1072 / SAD3569	Δ*dprA*::*Cm*^{R}, P_{tac}-*tfoX*, Δ*luxO*, *lacZ*::*lacI*^{q}, *pilA*^{S67C}, Δ*VC1807*::*Zeo*^{R}	Fig. 5	Δ*dprA* for NT frequency assays
TND4543 / SAD3570	Δ*recA*::*Spec*^{R}, P_{tac}-*tfoX*, Δ*luxO*, *lacZ*::*lacI*^{q}, *pilA*^{S67C}, Δ*VC1807*::*Zeo*^{R}	Fig. 5	Δ*recA* for NT frequency assays
TND4542 / SAD3571	Δ*recA*::*Spec*^{R}, Δ*sbcB*::*Tm*^{R}, P_{tac}-*tfoX*, Δ*luxO*, *lacZ*::*lacI*^{q}, *pilA*^{S67C}, Δ*VC1807*::*Zeo*^{R}	Fig. 5	Δ*sbcB* Δ*recA* for NT frequency assays
TND4667 / SAD3622	Δ*VCA0692*::*Carb*^{R}-P_{native}-*recA*, Δ*recA*::*Spec*^{R}, P_{tac}-*tfoX*, Δ*luxO*, *lacZ*::*lacI*^{q}, *pilA*^{S67C}, Δ*VC1807*::*Zeo*^{R}	Fig. 5	Δ*recA VCA0692*::*recA* for NT frequency assays
TND4663 / SAD3623	Δ*lacZ*::*Cm*^{R}-P_{native}-*sbcB*, Δ*sbcB*::*Tm*^{R}, P_{tac}-*tfoX*, Δ*luxO*, *pilA*^{S67C}, Δ*VC1807*::*Zeo*^{R}	Fig. 5	Δ*sbcB lacZ*::*sbcB* for NT frequency assays
TND4668 / SAD3624	Δ*VCA0692*::*Carb*^{R}-P_{native}-*recA*, Δ*recA*::*Spec*^{R}, Δ*sbcB*::*Tm*^{R}, P_{tac}-*tfoX*, Δ*luxO*, *lacZ*::*lacI*^{q}, *pilA*^{S67C}, Δ*VC1807*::*Zeo*^{R}	Fig. 5	Δ*sbcB* Δ*recA VCA0692*::*recA* for NT frequency assays
TND4666 / SAD3625	Δ*lacZ*::*Cm*^{R}-P_{native}-*sbcB*, Δ*sbcB*::*Tm*^{R}, Δ*recA*::*Spec*^{R}, Δ*sbcB*::*Tm*^{R}, P_{tac}-*tfoX*, Δ*luxO*, *pilA*^{S67C}, Δ*VC1807*::*Zeo*^{R}	Fig. 5	Δ*sbcB* Δ*recA lacZ*::*sbcB* for NT frequency assays
TND4338 / SAD3572	Δ*lacZ*::*Spec*^{R}-P_{native}-*sbcB*^{D13A,E15A}, Δ*sbcB*::*Tm*^{R}, P_{tac}-*tfoX*, Δ*luxO*, *pilA*^{S67C}, Δ*VC1807*::*Zeo*^{R}	Fig. 6A	Δ*sbcB lacZ*::*sbcB** for NT frequency assays
TND4407 / SAD3573	Δ*lacZ*::*Spec*^{R}-P_{native}-*sbcB*$_{E.coli}$, Δ*sbcB*::*Tm*^{R}, P_{tac}-*tfoX*, Δ*luxO*, *pilA*^{S67C}, Δ*VC1807*::*Zeo*^{R}	Fig. 6A	Δ*sbcB lacZ*::*sbcB*$_{Ec}$ for NT frequency assays
TND4472 / SAD3574	Δ*lacZ*::*Spec*^{R}-P_{native}-*sbcB*$_{E.coli}^{D15A,D17A}$, Δ*sbcB*::*Tm*^{R}, P_{tac}-*tfoX*, Δ*luxO*, *pilA*^{S67C}, Δ*VC1807*::*Zeo*^{R}	Fig. 6A	Δ*sbcB lacZ*::*sbcB*$_{Ec}$* for NT frequency assays
TND0111 / SAD1505	Δ*sbcB*::*Kan*^{R}	Fig. 6B	Δ*sbcB* for spontaneous Rif^R mutant frequency assays
TND4599 / SAD3575	Δ*recJ*::*Zeo*^{R}, Δ*exoVII*::*Carb*^{R}, Δ*exoIX*::Kan^R	Fig. 6B	Δ*recJ* Δ*exoVII* Δ*exoIX* for spontaneous Rif^R mutant frequency assays
TND4605 / SAD3576	Δ*sbcB*::*Tm*^{R}, Δ*recJ*::*Zeo*^{R}, Δ*exoVII*::*Carb*^{R}, Δ*exoIX*::Kan^R	Fig. 6B	Δ*sbcB* Δ*recJ* Δ*exoVII* Δ*exoIX* for spontaneous Rif^R mutant frequency assays
TND4606 / SAD3577	Δ*lacZ*::*Spec*^{R}-P_{native}-*sbcB*, Δ*sbcB*::*Tm*^{R}, Δ*recJ*::*Zeo*^{R}, Δ*exoVII*::*Carb*^{R}, Δ*exoIX*::Kan^R	Fig. 6B	Δ*sbcB* Δ*recJ* Δ*exoVII* Δ*exoIX* *lacZ*::*sbcB* for spontaneous Rif^R mutant frequency assays

TND4607 / SAD3578	Δ*lacZ*::*Spec*^{R}-P_{native}-*sbcB*^{D13A,E15A}, Δ*sbcB*::*Tm*^{R}, Δ*recJ*::*Zeo*^{R}, Δ*exoVII*::*Carb*^{R}, Δ*exoIX*::Kan^R	Fig. 6B	Δ*sbcB* Δ*recJ* Δ*exoVII* Δ*exoIX* *lacZ::sbcB** for spontaneous Rif^R mutant frequency assays
TND4288 / SAD3626	Δ*lacZ*::*Spec*^{R}-P^{tac}-*sbcB*, P_{tac}-*tfoX*, Δ*luxO*, *lacZ*::*lacI*^{q}, *pilA*^{S67C}, Δ*VC1807::Zeo*^{R}	Fig. 6C	P_{tac}-*sbcB* for ssDNA exonuclease activity assays
TND4665 / SAD3627	Δ*lacZ*::*Spec*^{R}-P^{tac}-*sbcB*^{D13A,E15A}, P_{tac}-*tfoX*, Δ*luxO*, *lacZ*::*lacI*^{q}, *pilA*^{S67C}, Δ*VC1807::Zeo*^{R}	Fig. 6C	P_{tac}-*sbcB** for ssDNA exonuclease activity assays
TND0904 / SAD2436	*comEA-mCherry*, P_{tac}-*tfoX*, Δ*luxO*, *lacZ*::*lacI*^{q}, *pilA*^{S67C}, Δ*VC1807::Cm*^{R}	Fig. 2	parent for ComEA-mCherry localization assays
TND4170 / SAD3579	Δ*pilQ*::*Tet*^{R}, *comEA-mCherry*, P_{tac}-*tfoX*, Δ*luxO*, *lacZ*::*lacI*^{q}, *pilA*^{S67C}, Δ*VC1807::Cm*^{R}	Fig. 2	Δ*pilQ* for ComEA-mCherry localization assays
JLC645 / SAD3580	Δ*comEC*::*Kan*^{R}, *comEA-mCherry*, P_{tac}-*tfoX*, Δ*luxO*, *lacZ*::*lacI*^{q}, *pilA*^{S67C}, Δ*VC1807::Cm*^{R}	Fig. 2	Δ*comEC* for ComEA-mCherry localization assays
TND4173 / SAD3581	Δ*sbcB::Tm*^{R}, *comEA-mCherry*, P_{tac}-*tfoX*, Δ*luxO*, *lacZ*::*lacI*^{q}, *pilA*^{S67C}, Δ*VC1807::Cm*^{R}	Fig. 2	Δ*sbcB* for ComEA-mCherry localization assays
SAD1063	*Kan*^{R}*-Ptac-tfoX*, Δ*mutS*	Fig. 3B-C	parent for *in vivo* branch migration assays
TND2585 / SAD3582	Δ*sbcB*::*Spec*^{R}, *Kan*^{R}*-Ptac-tfoX*, Δ*mutS*	Fig. 3B-C	Δ*sbcB* for *in vivo* branch migration assays
SAD1071	Δ*comM*::*Spec*^{R}, *Kan*^{R}*-Ptac-tfoX*, Δ*mutS*	Fig. 3B-C	Δ*comM* for *in vivo* branch migration assays
TND1474 / SAD3583	Δ*lacZ*::P_{lac}-*CFP-parB*$_{P1}$-*mCherry-parB*$_{MT1}$ *Zeo*^{R}, *0.11Mbp*::*parS*$_{MT1}$, *GFP-comM*, P_{tac}-*tfoX*, Δ*luxO*, *pilA*^{S67C}, Δ*VC1807::Cm*^{R}	Fig. 4A-C	parent for GFP-ComM localization assays
TND1509 / SAD3584	Δ*sbcB*::*Tm*^{R}, Δ*lacZ*::P_{lac}-*CFP-parB*$_{P1}$-*mCherry-parB*$_{MT1}$ *Zeo*^{R}, *0.11Mbp*::*parS*$_{MT1}$, *GFP-comM*, P_{tac}-*tfoX*, Δ*luxO*, *pilA*^{S67C}, Δ*VC1807::Cm*^{R}	Fig. 4B-C	Δ*sbcB* for GFP-ComM localization assays
SAD3547	*E. coli* TG1, pKT25-*sbcB*^{D13A,E15A}	Fig. 7	Harbors theT25-SbcB* vector for BACTH analysis
SAD3549	*E. coli* TG1, pKNT25-*sbcB*^{D13A,E15A}	Fig. 7	Harbors the SbcB*-T25 vector for BACTH analysis
SAD3558	*E. coli* TG1, pUT18C-*sbcB*^{D13A,E15A}	Fig. 7	Harbors the T18-SbcB* vector for BACTH analysis
SAD3551	*E. coli* TG1, pUT18-*sbcB*^{D13A,E15A}	Fig. 7	Harbors the SbcB*-T18 vector for BACTH analysis
TMN0416 / SAD3585	*E. coli* TG1, pUT18C-*recA*	Fig. 7	Harbors the T18-RecA vector for BACTH analysis
TMN0419 / SAD3587	*E. coli* TG1, pUT18-*recA*	Fig. 7	Harbors the RecA-T18 vector for BACTH analysis
TMN0417 / SAD3586	*E. coli* TG1, pUT18C-*dprA*	Fig. 7	Harbors the T18-DprA vector for BACTH analysis
TMN0420 / SAD3588	*E. coli* TG1, pUT18-*dprA*	Fig. 7	Harbors the DprA-T18 vector for BACTH analysis
SAD2240	*E. coli* TG1, pUT18C	Fig. 7	Harbors the T18 empty vector for BACTH analysis
SAD2241	*E. coli* TG1, pUT18C-zip	Fig. 7	Harbors the T18-zip vector for BACTH analysis
SAD2238	*E. coli* TG1, pKT25-zip	Fig. 7	Harbors the T25-zip vector for BACTH analysis

*Multiple Strain# designations indicate that the strain was stocked in two independent strain collections.
