# Supplementary material for: SbcB facilitates natural transformation in Vibrio cholerae in an exonuclease-independent manner
Source: J Bacteriol. 2024 Dec 13;207(1):e00419-24. doi: 10.1128/jb.00419-24 (PMC11784430; doi:10.1128/jb.00419-24)
Supplement: Table S2 — Primers used in this study. [file jb.00419-24-s0003.pdf]

Table S2 – *Primers used in this study*

Primer*	Sequence (5' → 3')	Description
BBC688	TGGATGAGTGCTAAATGATGC	Δ*sbcB* (*i.e.*, ΔVC1234) F1
BBC689	gtcgacggatccccggaatCATGTGGCTTACCAAATCGC	Δ*sbcB* (*i.e.*, ΔVC1234) R1
BBC690	gaagcagctccagcctacaTGCTAACATCGTTTATTTTTACTTGC	Δ*sbcB* (*i.e.*, ΔVC1234) F2
BBC691	AACATGGTAAACAGCACCATC	Δ*sbcB* (*i.e.*, ΔVC1234) R2
DOG0582	ACATCTGGCAGATCTGATTTCAC	Δ*pilQ* (*i.e.*, ΔV2630) F1
DOG0583	gtcgacggatccccggaatCAACCGATTATTTTAAAGCCAGCTTG	Δ*pilQ* (*i.e.*, ΔV2630) R1
DOG0584	gaagcagctccagcctacaTAACTTAGCGTGTGCGTAACAAG	Δ*pilQ* (*i.e.*, ΔV2630) F2
DOG0585	TTGTGTTTGCTCTAACGTTTGC	Δ*pilQ* (*i.e.*, ΔV2630) R2
BBC411	CAAGATTGGCAATGGAGCGAC	Δ*comEC* (*i.e.*, ΔVC1879) F1
BBC412	gtcgacggatccccggaatCATAATCCCGTATGCCAAGAAAATTC	Δ*comEC* (*i.e.*, ΔVC1879) R1
BBC546	gaagcagctccagcctacaATGACCGATACTCAGTCTAACC	Δ*comEC* (*i.e.*, ΔVC1879) F2
BBC414	TCATCCATGCTCATGCTGCG	Δ*comEC* (*i.e.*, ΔVC1879) R2
ABD820	CGCTCTTATCTGCTTGGATAATGG	Δ*dprA* (*i.e.*, ΔVC0048) F1
ABD998	gtcgacggatccccggaatCATTAACTGGCATCATCAACC	Δ*dprA* (*i.e.*, ΔVC0048) R1
ABD999	gaagcagctccagcctacaTAGCTATGATGATGGATATTTTGATG	Δ*dprA* (*i.e.*, ΔVC0048) F2
ABD996	GGTTTGGTTATCCGGATCACAC	Δ*dprA* (*i.e.*, ΔVC0048) R2
ABD855	CATGAATCACTTTGGCATGAGG	Δ*comM* (*i.e.*, ΔVC0032) F1
ABD856	gtcgacggatccccggaatCATTGCTTCCCTTAGTATTTGATC	Δ*comM* (*i.e.*, ΔVC0032) R2
ABD857	gaagcagctccagcctacaTAGTACTCTGACCTGCAGAGTTC	Δ*comM* (*i.e.*, ΔVC0032) F2
ABD858	AAATTCCAGAAAAACCACGTC	Δ*comM* (*i.e.*, ΔVC0032) R2
BBC678	TGGATGCCAATCAACATTCG	Δ*recJ* (*i.e.*, ΔVC2417) F1
BBC679	gtcgacggatccccggaatCATACTGTGACAGGCCAAAG	Δ*recJ* (*i.e.*, ΔVC2417) R1
BBC680	gaagcagctccagcctacaGAAGCGAAATGATTGAAAACAACG	Δ*recJ* (*i.e.*, ΔVC2417) F2
BBC681	GATCGCATCCACAATGTTAGC	Δ*recJ* (*i.e.*, ΔVC2417) R2
DOG0185	TTCACTTCACCCAGTACACGC	Δ*exoVII* (*i.e.*, ΔVC0766) F1
DOG0186	gtcgacggatccccggaatCAACGCTGATTCCTCAGACG	Δ*exoVII* (*i.e.*, ΔVC0766) R1
DOG0187	gaagcagctccagcctacaTTAATGGATGGTGAGATTCTCTC	Δ*exoVII* (*i.e.*, ΔVC0766) F2
DOG0188	AGTTTGTAGAGGTTGTTATGGTAC	Δ*exoVII* (*i.e.*, ΔVC0766) R2
DOG0190	AGAAGAACTCTGTTTTGCATTAGAAC	Δ*exoIX* (*i.e.*, ΔVC0898) F1
DOG0191	gtcgacggatccccggaatCAAGCGACGAGTTCATGCTTG	Δ*exoIX* (*i.e.*, ΔVC0898) R1
DOG0192	gaagcagctccagcctacaTAAATCCCCTCTGATTAGCATC	Δ*exoIX* (*i.e.*, ΔVC0898) F2
DOG0193	TTAACCCTGACGTGACCGTG	Δ*exoIX* (*i.e.*, ΔVC0898) R2
BBC5040	CAAGTagCGTAcgCGAAAAAGAAGAATGTGGGCTGGTGTTCTTGCT	$sbcB^{D13AE15A}$ R1
BBC5041	TTCTTTTTCGcgTACGctACTTGGGGAGTCAATCCCGCAAAAGATC	$sbcB^{D13AE15A}$ F2

BBC4977	gcctggagtgcctaatgagGCTGAACAAATAATGGGATAGC	Amplify *sbcB* to clone at an ectopic locus with its native promoter intact F
BBC3101	tgtaggctggagctgcttcTTAGCAAGCGAGTTTTTCCACG	Amplify *sbcB* to clone at an ectopic locus with its native or P_{tac} promoter intact R
BBC5065	tttagcgatttggtaagccacATGATGAATGACGGTAAGCAAC	Amplify *E. coli* sbcB to clone at an ectopic locus F
BBC5066	tgtaggctggagctgcttcTTAGACAATCTCTTCCGCGTAC	Amplify *E. coli* sbcB to clone at an ectopic locus R
BBC3100	caatttcacacaggatcccgggaggaggtAGCCACATGCAGCAAGAACAC	Amplify *sbcB* to clone at an ectopic locus under the control of P_{tac} F
BBC5203	gcctggagtgcctaatgagTTCAAGGTTGGCTCAAAGTGG	Amplify *recA* to clone at an ectopic locus with its native promoter intact F
BBC655	tgtaggctggagctgcttcTTAAAACTCTTCTGGCACCGC	Amplify *recA* to clone at an ectopic locus with its native promoter intact R
BBC5145	AGGTagCGTAcgCGTGAAACAAAAAGGTAGATTGTTGCTTACCGTC	*E. coli* $sbcB^{D15AE17A}$ R1
BBC5146	TTGTTTCACGcgTACGctACCTTTGGCACGCACCCCGCGTTAGATC	*E. coli* $sbcB^{D15AE17A}$ F2
BBC5195	gcaggtggagcaggtggaCAGCAAGAACACCAGCCCAC	Amplify *sbcB** for TXX-*sbcB** or *sbcB**-TXX for BACTH F
BBC3103	tgtaggctggagctgcttcTTAGGCTGCGATTAATAGCTCTTTAG	Amplify *sbcB** for TXX-*sbcB** for BACTH R
BBC5196	tccaccacttccacctgcGCAAGCGAGTTTTTCCACGTAATGG	Amplify *sbcB** for *sbcB**-TXX for BACTH R
TMN0198	gcaggtggagcaggtggaGACGAGAATAAACAGAAGGCAC	Amplify *recA* for TXX-*recA* or *recA*-TXX for BACTH F
TMN0199	tgtaggctggagctgcttcTTAAAACTCTTCTGGCACCGC	Amplify *recA* for TXX-*recA* for BACTH R
TMN0200	tccaccacttccacctgcAAACTCTTCTGGCACCGC	Amplify *recA* for *recA*-TXX for BACTH R
TMN0210	gcaggtggagcaggtggaAAAGATCAGGATTTAGCGGCATG	Amplify *dprA* for TXX-*dprA* or *dprA*-TXX for BACTH F
TMN0211	tgtaggctggagctgcttcCTAGCCTCTCCCCTTTCTAATATAGC	Amplify *dprA* for TXX-*dprA* for BACTH R
TMN0212	tccaccacttccacctgcGCCTCTCCCCTTTCTAATATAGCC	Amplify *dprA* for *dprA*-TXX for BACTH R
PP327	IR800CW-AAATTTGGCTATTGATAGTGCTCATTG	IR800 ssDNA oligo used for ssDNA exonuclease activity assays

*Lower case nucleotides on the 5' end demarcate overlap sequences for SOE PCR. While internal lower case nucleotides demarcate point mutations.
